# Pulmonary Metastases From a Long-Standing Untreated Facial Basal Cell Carcinoma: A Rare Case Report

**DOI:** 10.7759/cureus.112946

**Published:** 2026-07-19

**Authors:** Glorimar Santiago Rivera, Aidimar Rodriguez Fuentes, Carolina Gaud Rodriguez, Jessyca C Santiago-Morales, María J Marcos Martínez

**Affiliations:** 1 Internal Medicine, University of Medicine and Health Sciences, Basseterre, KNA; 2 Family Medicine, University of Medicine and Health Sciences, Basseterre, KNA; 3 Internal Medicine, San Juan City Hospital, San Juan, PRI; 4 Pathology and Laboratory Medicine, University of Puerto Rico, Medical Sciences Campus, San Juan, PRI; 5 Pathology, Administración de Servicios Médicos de Puerto Rico, Medical Center of Puerto Rico, San Juan, PRI

**Keywords:** advanced basal cell carcinoma, basal cell carcinoma, case report, immunohistochemistry, metastatic basal cell carcinoma, neglected basal cell carcinoma, pulmonary metastasis

## Abstract

Basal cell carcinoma (BCC) is the most common cutaneous malignancy and is typically characterized by slow growth, local tissue invasion, and an excellent prognosis when treated early. Although metastatic disease is exceedingly uncommon, the lungs are among the most frequently reported sites of distant spread. We report the case of a 64-year-old man with a 15-year history of untreated facial BCC who presented with a painful ulcerated lesion associated with chronic purulent drainage, progressive visual decline in the left eye, difficulty with mastication, and unintentional weight loss of approximately 50 lbs over two years. Physical examination revealed a destructive 13 × 15 cm ulcerative neoplastic lesion involving the left hemiface, temporal region, occipital region, auricular area, and ipsilateral neck, with extensive tissue destruction, exposed bone, necrosis, foul-smelling purulent drainage, and findings concerning for superimposed soft tissue infection. Imaging demonstrated extensive craniofacial destruction involving the temporal bone, zygomatic arch, and mandibular condyle, while chest computed tomography revealed multiple bilateral spiculated pulmonary nodules measuring up to 2.5 cm. Histopathologic examination of the facial lesion confirmed BCC, and CT-guided biopsy of a pulmonary nodule established the diagnosis of metastatic BCC. The patient received broad-spectrum intravenous antibiotics for the superimposed infection and was evaluated for systemic therapy with vismodegib or cemiplimab. This case illustrates how prolonged treatment delay can transform a highly curable malignancy into locally destructive and metastatic disease.

## Introduction

Basal cell carcinoma (BCC) is the most common cutaneous malignancy and one of the most frequently diagnosed human cancers. It arises from keratinocytes of the basal layer of the epidermis and most commonly develops in chronically sun-exposed areas of the skin. BCC is typically characterized by slow growth, local tissue invasion, and a favorable prognosis when diagnosed and treated at an early stage [1]. The majority of BCC lesions can be successfully managed with local therapies, including surgical excision and Mohs micrographic surgery [2].

Despite its high prevalence, metastatic basal cell carcinoma (mBCC) remains exceedingly uncommon, with a reported incidence ranging from 0.003% to 0.55% [3]. When advanced disease does occur, it is often associated with delayed diagnosis, barriers to healthcare access, and prolonged treatment avoidance [4]. Factors associated with aggressive behavior and metastatic progression include prolonged untreated disease, large tumor size, invasion of deep structures, and tumors arising in the head and neck region [5,6]. In patients with these high-risk features, additional imaging and staging evaluation may be warranted to assess the extent of local invasion and identify potential regional or distant metastases [5].

Recent advances in systemic therapy have significantly changed the management of advanced BCC. Hedgehog pathway inhibitors, including vismodegib, and, more recently, immune checkpoint inhibitors such as cemiplimab have expanded treatment options for patients with unresectable locally advanced or metastatic disease [6,7].

We report a rare case of a long-standing, untreated facial BCC associated with extensive craniofacial destruction and biopsy-confirmed pulmonary metastases. This case demonstrates how prolonged neglect of an otherwise highly curable malignancy can culminate in extensive local destruction and metastatic disease, highlighting the importance of early recognition and timely intervention.

## Case presentation

A 64-year-old man with approximately 40 years of occupational sun exposure as a house painter and a 20-pack-year smoking history (quit two years prior) presented to the ED after an episode of dizziness resulting in a fall with minor head trauma. His home medications included nabumetone 500 mg once daily and ketorolac 10 mg once daily for chronic pain. Family history was notable for paternal throat cancer; there was no known family history of skin cancer or hereditary cancer syndromes. During evaluation, he reported progressive enlargement of the facial lesion over several years, accompanied by chronic facial pain, intermittent purulent drainage, progressive visual decline in the left eye, difficulty with mastication due to pain, generalized weakness, and an unintentional weight loss of approximately 50 lbs over the preceding two years. He denied any prior treatment of the lesion, including surgical excision, chemotherapy, radiation therapy, or reconstructive procedures. He also reported housing instability following a conflict with his daughter, a social circumstance that may have further contributed to delays in seeking medical care.

Physical examination revealed a large, destructive ulcerative-neoplastic lesion involving the left hemiface, extending from the temporal region to the mandibular area and posteriorly to the occipital region and ipsilateral neck. The lesion measured approximately 13 × 15 cm and was associated with extensive tissue destruction, absence of the left auricle, exposed underlying bone, necrotic eschar, foul odor, and continuous purulent and serosanguineous drainage. Marked tenderness to palpation, facial asymmetry, and left lower eyelid ptosis secondary to tumor involvement were also noted (Figure 1).

**Figure 1:**
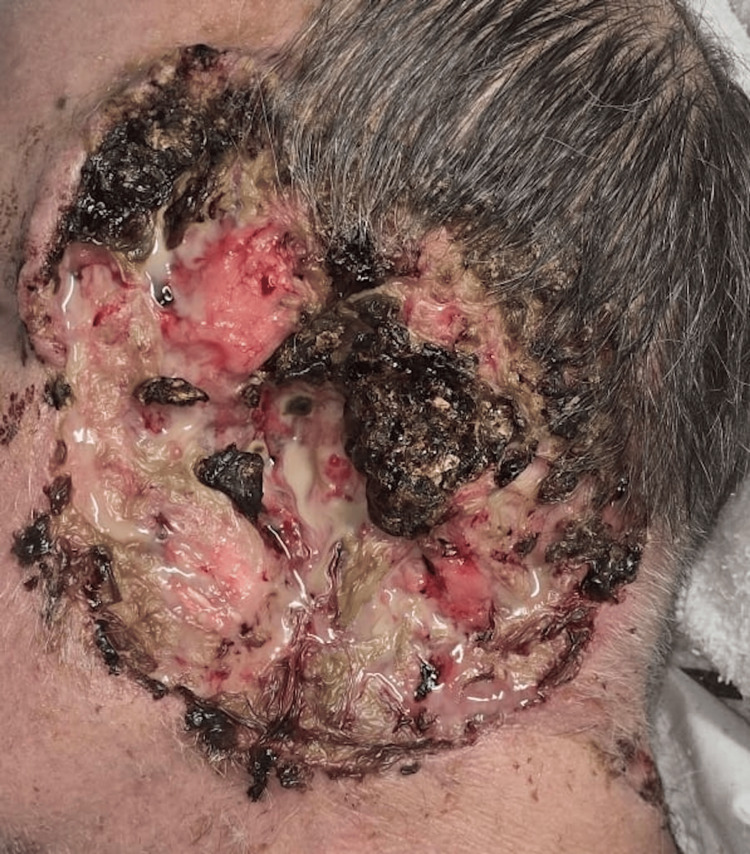
Clinical photograph of the left hemiface. This image demonstrates a large ulcerative neoplastic lesion involving the left hemiface, with extensive tissue destruction, necrosis, exposed bone, and complete loss of the left auricle at presentation.

An incisional skin biopsy of the facial lesion was performed. Pathologic examination of the facial lesion (Figure 2) demonstrated an infiltrative epithelial neoplasm composed of irregular nests and cords of atypical hyperchromatic basaloid cells. The tumor exhibited characteristic peripheral palisading, stromal retraction artifact, and mucinous material, findings consistent with BCC. Immunohistochemistry showed weak, patchy BCL-2 expression and focal Ep-CAM positivity. There was positivity for CK5/6 and p63, with focal epithelial membrane antigen (EMA) and chromogranin immunoreactivity. Tumor cells were negative for CK7, CK20, TTF-1, Napsin A, c-KIT (CD117), and synaptophysin.

**Figure 2:**
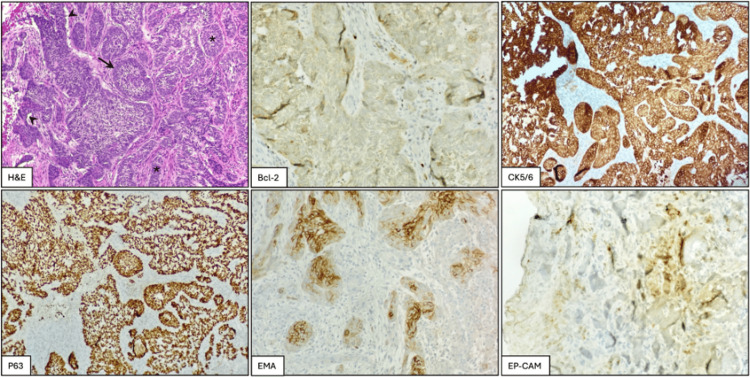
Histopathologic and immunohistochemical findings of the facial lesion. Microscopic examination (H&E, ×200) reveals an infiltrative epithelial neoplasm composed of irregular nests and cords of atypical hyperchromatic basaloid cells (asterisk). The tumor exhibits characteristic peripheral palisading (arrow) and stromal retraction artifact (arrowhead), findings consistent with basal cell carcinoma. Immunohistochemistry shows weak, patchy BCL-2 expression, diffuse CK5/6 and p63 positivity, and focal EMA and Ep-CAM positivity. BCL-2: B-cell lymphoma 2; Ep-CAM: Epithelial cell adhesion molecule.

Non-contrast CT of the head and contrast-enhanced CT of the maxillofacial region demonstrated extensive left-sided destructive facial changes involving the temporal bone, zygomatic arch, mandibular condyle, and adjacent soft tissues, resulting in marked facial deformity (Figure 3). No acute intracranial hemorrhage, mass effect, or midline shift was identified.

**Figure 3:**
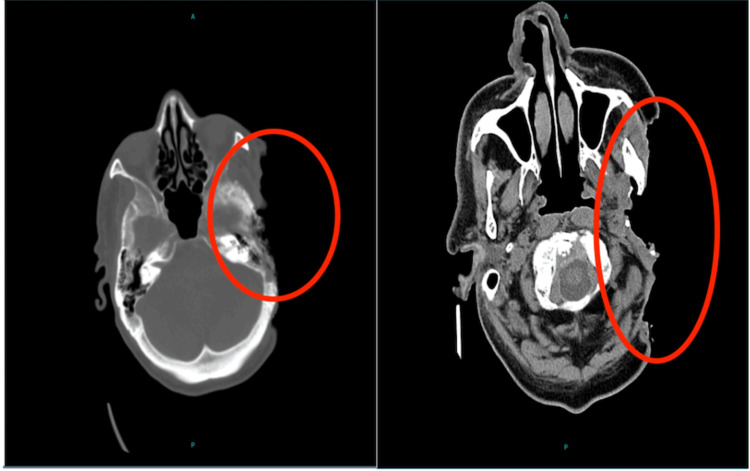
Axial CT images of the head and maxillofacial region. The images show extensive left-sided facial destruction with involvement of adjacent osseous structures, including the temporal bone, zygomatic arch, and mandibular condyle. Left: Contrast-enhanced CT image. Right: Non-contrast CT image.

Staging CT of the chest revealed multiple bilateral spiculated pulmonary nodules with small bilateral pleural effusions. The largest lesion was a peripheral left lower lobe pulmonary nodule measuring up to 2.5 cm, which was highly suspicious for metastatic disease (Figure 4). Pulmonology was consulted for evaluation of the pleural effusions. Bedside thoracic USG demonstrated small bilateral pleural effusions without an adequate pleural fluid pocket for safe thoracentesis. Therefore, thoracentesis was deferred, and the patient was managed conservatively with clinical monitoring. Contrast-enhanced CT of the abdomen and pelvis demonstrated no evidence of intra-abdominal or pelvic metastatic disease and no other acute abdominopelvic pathology. Laboratory evaluation was notable for microcytic anemia, thrombocytosis, hypoalbuminemia, and initial mild leukocytosis that improved following treatment (Table 1).

**Figure 4:**
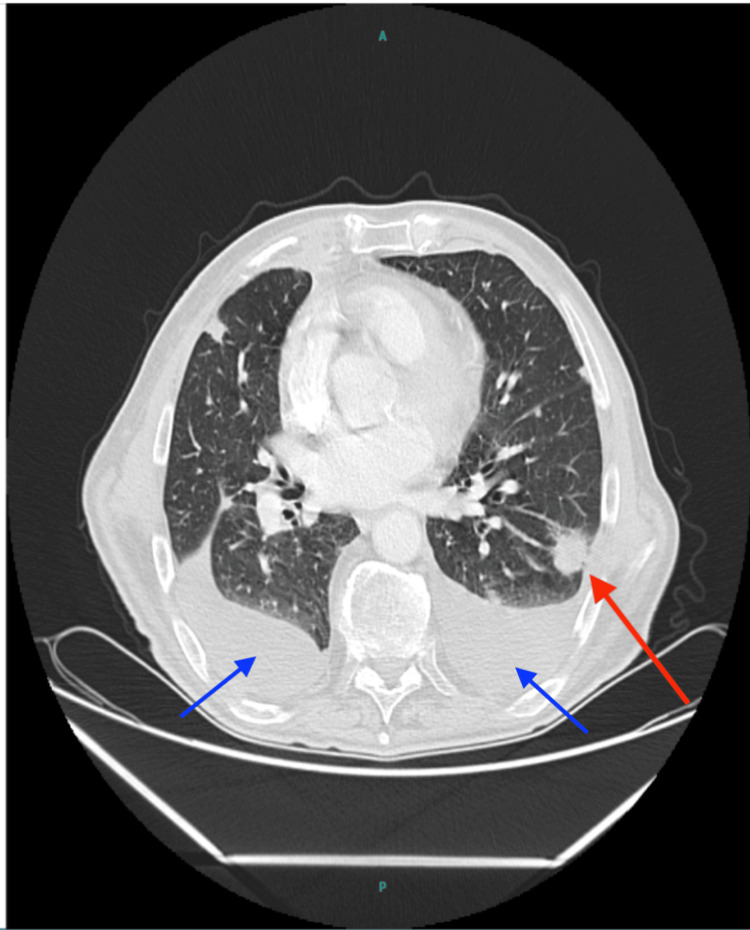
Contrast-enhanced CT image of the chest. The image demonstrates a spiculated left lower lobe pulmonary nodule measuring approximately 2.5 cm (red arrow), subsequently confirmed by CT-guided biopsy to represent metastatic basal cell carcinoma. Small bilateral pleural effusions are also visible (blue arrows).

**Table 1:** Laboratory findings during hospitalization. Laboratory values at hospital admission and after resolution of the superimposed soft tissue infection following intravenous antibiotic therapy. ↑ indicates values above the reference range, and ↓ indicates values below the reference range.

Laboratory test	Reference range	Admission	Post-treatment
WBC count (×10³/µL)	4.8-10.8	11.9 ↑	8.2
Hemoglobin (g/dL)	14.0-18.0	10.2 ↓	7.7 ↓
Platelet count (×10³/µL)	150-450	712 ↑	506 ↑
Sodium (mmol/L)	136-145	135 ↓	137
Potassium (mmol/L)	3.5-5.1	5.3 ↑	4.7
Creatinine (mg/dL)	0.70-1.20	1.1	0.82
Albumin (g/dL)	3.5-5.2	4	2.7 ↓
Total protein (g/dL)	6.4-8.3	7.6	5.3 ↓
Alkaline phosphatase (IU/L)	40-129	105	104

A CT-guided core needle biopsy of the left lower lobe pulmonary nodule was performed. Pathologic examination (Figure 5) demonstrated an infiltrative biphasic epithelial neoplasm composed of nests of atypical hyperchromatic basaloid cells with peripheral palisading, admixed with foci of squamous differentiation. Immunohistochemistry showed diffuse Ep-CAM, CK5/6, and p63 positivity, with focal chromogranin immunoreactivity. Tumor cells were negative for BCL-2, CK7, CK20, TTF-1, Napsin A, EMA, c-KIT, and synaptophysin. Histopathologic and immunohistochemical findings were consistent with mBCC, basosquamous variant. Primary lung adenocarcinoma, neuroendocrine carcinoma, and Merkel cell carcinoma were considered in the differential diagnosis and excluded primarily based on the immunohistochemical findings.

**Figure 5:**
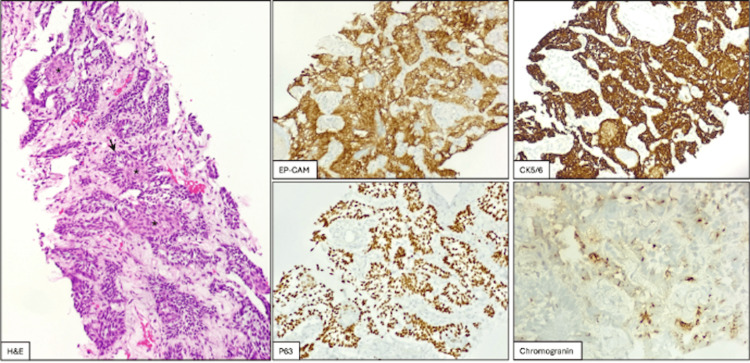
Histopathologic and immunohistochemical findings of the pulmonary core needle biopsy. Microscopic examination with H&E staining at ×200 magnification demonstrates an infiltrative biphasic epithelial neoplasm composed of nests of atypical hyperchromatic basaloid cells with peripheral palisading (arrow), admixed with foci of squamous differentiation (asterisk), consistent with metastatic basal cell carcinoma, basosquamous variant. Immunohistochemistry shows diffuse epithelial cell adhesion molecule (Ep-CAM), cytokeratin 5/6 (CK5/6), and tumor protein p63 (p63) positivity, with focal chromogranin immunoreactivity. Tumor cells are negative for B-cell lymphoma 2 (BCL-2), cytokeratin 7 (CK7), cytokeratin 20 (CK20), thyroid transcription factor 1 (TTF-1), Napsin A, epithelial membrane antigen (EMA), c-KIT (CD117), and synaptophysin, supporting the diagnosis of metastatic basal cell carcinoma.

The patient was treated with broad-spectrum IV antibiotics, including vancomycin and piperacillin-tazobactam, for a superimposed soft tissue infection. Laboratory findings during hospitalization are summarized in Table 1. Follow-up clinical examination demonstrated resolution of the superimposed infection with granulation tissue formation (Figure 6). Hematology/Oncology recommended outpatient systemic therapy with either vismodegib or cemiplimab following discharge. Pain management was optimized with tramadol, acetaminophen, and gabapentin, and Social Work was involved in discharge planning and coordination of follow-up care.

**Figure 6:**
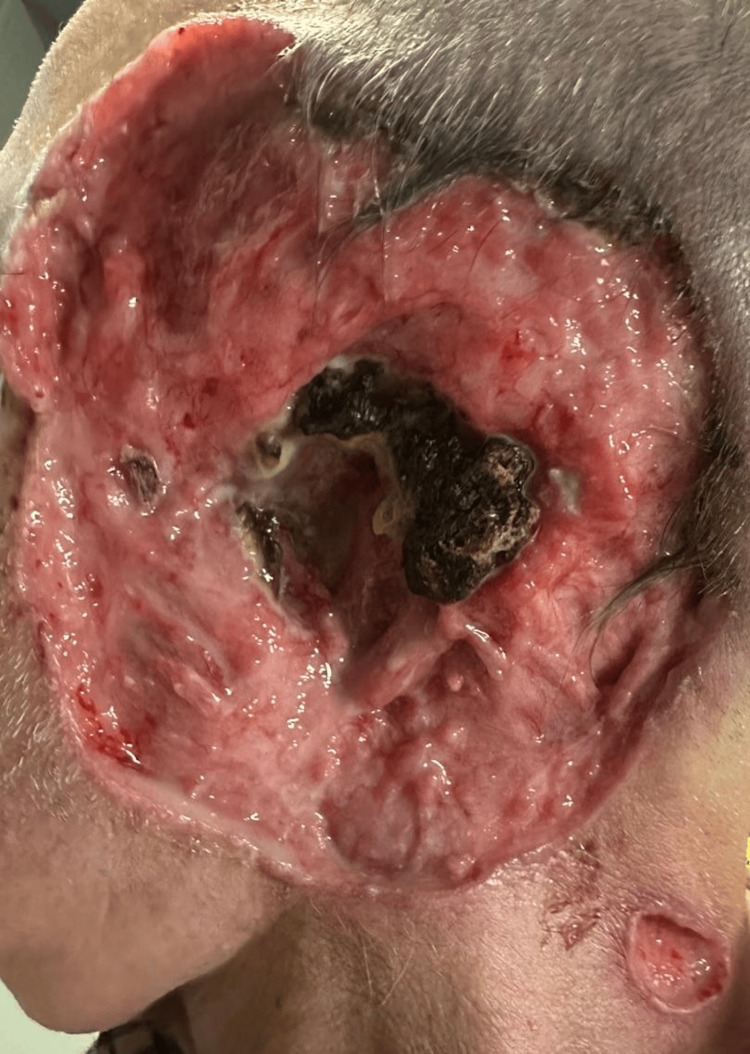
Clinical photograph of the hemiface. This image was obtained during hospitalization after completion of IV antibiotic therapy and demonstrates locally advanced basal cell carcinoma with resolution of the superimposed infection and granulation tissue formation.

## Discussion

BCC is a highly prevalent cutaneous malignancy that is typically associated with favorable clinical outcomes. Although BCC accounts for the majority of nonmelanoma skin cancers, metastatic disease remains exceedingly uncommon, with reported incidences ranging from 0.003% to 0.55% [3]. Pulmonary involvement is a recognized pattern of distant spread in mBCC and is thought to occur predominantly through hematogenous dissemination [4,7].

The present case is notable because it demonstrates biopsy-confirmed pulmonary metastases arising from a long-standing untreated facial BCC. While most BCCs remain confined to the skin and are successfully managed with local therapy, our patient exhibited several features associated with aggressive tumor behavior, including an untreated duration of approximately 15 years, a large tumor burden, a head and neck location, extensive invasion of adjacent soft tissue and osseous structures, and a basosquamous histopathologic variant. These characteristics have been consistently associated with an increased risk of advanced local invasion and metastatic progression in previous studies [4,5,8].

Another remarkable aspect of this case was the degree of local destruction. The lesion measured approximately 13 × 15 cm and was associated with loss of the left auricle, involvement of the temporal bone, zygomatic arch, and mandibular condyle, progressive visual impairment, chronic pain, difficulty with mastication, and substantial unintentional weight loss. Reports of giant and neglected BCCs have demonstrated that these tumors may produce significant morbidity even in the absence of distant metastases through progressive tissue destruction, chronic infection, nutritional compromise, and functional impairment [8]. Notably, lesions exceeding 10 cm in diameter have been associated with substantially increased risks of metastasis and disease-related mortality, emphasizing the significance of the advanced disease burden observed in our patient [8].

The social context of this case is also noteworthy. Giant and neglected BCCs are frequently associated with prolonged delays in seeking medical attention, low socioeconomic status, limited access to healthcare, and other barriers to treatment [8]. Similar circumstances were present in our patient, whose lesion progressed untreated for more than a decade before presentation. This observation highlights the potential influence of social determinants of health on disease progression and clinical outcomes.

Pulmonary involvement in our patient is consistent with previously reported patterns of metastatic BCC. Case series have shown that, although lymph nodes are often the initial site of spread, the lungs represent one of the most frequent and clinically significant sites of distant metastasis, with patients typically developing metastases years after initial diagnosis and receiving multimodal therapy, including surgery, chemotherapy, and radiotherapy [9]. Similarly, in a clinicopathologic series of 22 patients with metastatic BCC, 10 patients had biopsy-confirmed pulmonary metastases, making the lungs the most common site of distant metastatic disease [10]. The presence of multiple bilateral pulmonary nodules in our patient, subsequently confirmed by CT-guided biopsy, therefore reflects a recognized but uncommon pattern of advanced disease. Histopathologic confirmation of pulmonary involvement further strengthens the diagnosis and underscores the aggressive nature of this presentation.

Most BCC lesions are effectively treated with local therapies, including surgical excision and Mohs micrographic surgery. Mohs micrographic surgery remains the preferred treatment for high-risk lesions, providing excellent long-term disease control, with reported 5-year cure rates exceeding 99% for primary BCCs and 96% for recurrent BCCs and low recurrence rates of approximately 3.1% for primary BCCs and 5.3% for recurrent BCCs [2].

Alternative treatment modalities, including radiation therapy, may be considered for patients who are not surgical candidates or who present with unresectable disease, whereas topical and destructive therapies are generally reserved for lower-risk lesions [4]. In patients with locally advanced, symptomatic head and neck non-melanoma skin cancers, palliative radiotherapy has demonstrated high rates of symptom palliation, including significant improvement in pain, while maintaining an acceptable toxicity profile and preserving quality of life for most patients [11]. Therefore, radiotherapy may represent an important component of multidisciplinary care for selected patients, particularly when surgery is not feasible or when rapid local symptom control is a priority.

Management of advanced BCC has evolved substantially with the introduction of systemic therapies. Hedgehog pathway inhibitors remain the standard first-line treatment for unresectable or metastatic disease. In the pivotal ERIVANCE trial, vismodegib achieved an objective response rate of approximately 30% in patients with metastatic BCC and a median progression-free survival of 9.3 months. For patients whose disease progresses during treatment with, or who are intolerant to, Hedgehog pathway inhibitors, PD-1 blockade with cemiplimab has emerged as an effective second-line option. In the final phase II analysis, cemiplimab demonstrated an objective response rate of 22%, a disease control rate of 63%, and durable responses, with a median duration of response that was not reached, supporting its role in patients with advanced metastatic BCC after Hedgehog pathway inhibitor therapy. These findings support the recommendation of vismodegib as first-line systemic therapy and cemiplimab as an effective subsequent option for patients with advanced metastatic BCC, consistent with the treatment recommendation made for our patient following discharge [4,7].

This case illustrates how prolonged treatment delay can transform one of the most curable human malignancies into a devastating disease characterized by extensive craniofacial destruction, profound functional impairment, and rare distant metastasis. Beyond documenting an uncommon presentation of pulmonary mBCC, this case underscores the critical importance of addressing barriers to early diagnosis, timely intervention, and access to specialized oncologic care in preventing advanced disease and its associated morbidity.

## Conclusions

Although BCC is typically associated with an excellent prognosis, delayed treatment may result in severe local destruction and, in rare cases, metastatic disease. Early recognition and prompt intervention remain essential for preventing advanced disease and improving patient outcomes.
